# Mental Well-Being during COVID-19: A Cross-Sectional Study of Fly-In Fly-Out Workers in the Mining Industry in Australia

**DOI:** 10.3390/ijerph182212264

**Published:** 2021-11-22

**Authors:** Bernard Yeboah-Asiamah Asare, Elizabeth Thomas, Jacquita S. Affandi, Myles Schammer, Paul Brown, Matthew Pilbeam, Chris Harris, Chris Ellison, Dominika Kwasnicka, Daniel Powell, Christopher M. Reid, Suzanne Robinson

**Affiliations:** 1 Curtin School of Population Health, Curtin University, Kent Street, Bentley 6102, Australia; libby.thomas@curtin.edu.au (E.T.); jacquita.affandi@curtin.edu.au (J.S.A.); christopher.reid@curtin.edu.au (C.M.R.); suzanne.robinson@curtin.edu.au (S.R.); 2 Institute of Applied Health Sciences, University of Aberdeen, Aberdeen AB25 2ZD, UK; daniel.powell@abdn.ac.uk; 3 Mineral Resources Limited, Applecross 6153, Australia; myles.schammer@mrl.com.au (M.S.); Paul.Brown@mrl.com.au (P.B.); Matthew.Pilbeam@mrl.com.au (M.P.); chris.harris@mrl.com.au (C.H.); chris.ellison@mrl.com.au (C.E.); 4 Faculty of Psychology, SWPS University of Social Sciences and Humanities, Aleksandra Ostrowskiego, 53-238 Wroclaw, Poland; dkwasnicka@swps.edu.pl; 5 NHMRC CRE in Digital Technology to Transform Chronic Disease Outcomes, Melbourne School of Population and Global Health, University of Melbourne, Melbourne 3000, Australia; 6 Rowett Institute, University of Aberdeen, Aberdeen AB25 2ZD, UK

**Keywords:** COVID-19, fly-in fly-out, FIFO, mental well-being, mental health, mining, Australia

## Abstract

Coronavirus disease 2019 (COVID-19) has devastated the world, and its mental health impact has been recognized in the general population. However, little is known about the mental health impact of COVID-19 on fly-in fly-out (FIFO) workers, who are flown to temporarily stay and work in remote areas, during this pandemic. This study examined the mental well-being of FIFO workers in the mining industry during COVID-19 restrictions in Western Australia. An online survey was conducted between May to November 2020 among (*N* = 842) FIFO workers who underwent COVID-19 screening at a large mining company in Western Australia. The mental well-being score among workers was higher than population norms. One-way ANOVA with Bonferroni post-hoc tests showed significant differences in mental well-being by age, being placed under travel quarantine, undertaking self-isolation, impact of social distance guidelines, and experience of COVID-19 related symptoms. Multiple linear regression analysis showed workers who were younger, placed under travel quarantine and experienced two or more COVID-19 related symptoms were more likely to have worse mental well-being. Acknowledging the negative emotions and distress experiences among the vulnerable groups could help in providing suitable support to help lessen these negative experiences in FIFO workers.

## 1. Introduction

Coronavirus disease (COVID-19) has devastated lives and economies around the world; since its detection in December 2019 [1]. In March 2020, the World Health Organization (WHO) declared COVID-19 as a pandemic having infected over 118,000 people and caused 4291 deaths in 114 countries [2]. As of 4 October 2021, there were 234,609,003 established COVID-19 cases, and 4,797,368 lives lost around the world [3]. In January 2020, Australia recorded its first COVID-19 case [4], and now has recorded 113,411 confirmed cases with 1344 deaths as of 4 October 2021 [5].

This public health emergency necessitated extraordinary measures to be taken in order to limit the spread or transmission of the virus in the general population across the world. These measures, namely social distancing, aimed at limiting close contact and onward person-to-person contact transmission of the virus [6] which included travel bans, lockdown and movement restrictions, self-isolation and/or quarantine of cases, and closures of schools, workplaces, and social/community gatherings [6,7]. In Australia, COVID-19 restrictions and lockdown measures were introduced from 21st March 2020 [8] and by 31st March 2020, Stage 3 COVID-19 restrictions were in place, where stay at home orders were mandatory, except for essential workers (medical care and caregiving), and members of the public were only allowed out of the home to shop for basic supplies, to exercise, and for work that could not be done from home [9]. These restrictions lasted until 15th May 2020 when some Australian states began easing restrictions on public gathering and non-essential services [8].

Social distancing measures have proven to be effective in reducing the spread or transmission of the virus [7,10,11] and associated deaths [11]. However, the negative impact of such measures on mental health have been recognized [8,12] including increases in psychological distress, loneliness, and a general negative impact on psychosocial well-being [8], as they unavoidably interrupt the normal/usual social practices, such as access to social support systems, everyday interactions, and social impacts on coping, that benefit mental health [13]. Furthermore, associated factors of pandemic separations and restrictions such as the fear of infection or infecting loved ones or others, longer durations of social isolation measures, limited access to basic supplies, the lack of clarity around guidelines and purposes of social distancing measures, and stigmatisation can cause frustration and anxiety in people placed under quarantine [14]. As such, COVID-19 present an important short- and long-term mental health problem to populations around the world [15].

The mental health impact of COVID-19 pandemic has been documented in the general population globally [9,15,16,17]. For instance, a systematic review of 19 studies found that depression and anxiety levels were higher and psychological well-being was poorer among the general population globally compared to periods prior to COVID-19 [18]. Other systematic reviews found the general population in China experienced psychological distress including anxiety, depression, stress and posttraumatic symptoms associated with COVID-19 outbreak [19,20]. A longitudinal study has also found increases in suicidal ideation, suicide attempts and self-harm over time from 31 March to 19 April 2021 among the general adult population in the United Kingdom [21]. In the United States, the proportion of emergency department visits for mental health conditions and suicide attempts were markedly higher during the COVID-19 pandemic than the same period in 2019 [22].

In Australia, several studies have also highlighted the negative impact of COVID-19 on mental health in individuals and the general populace [9,15,23,24,25]. A national study found higher prevalence of restlessness, nervousness, hopelessness and worthlessness among the general populace than previously recorded [25]. Another national study also found elevated prevalence of depression and anxiety, feelings of hurting oneself, and irritability [24]. Other studies have examined the negative influence of COVID-19 on the mental health in specific populations such as healthcare workers and ‘other essential workers’ such as police officers, logistics and transport and retail hospitality workers [9] and the general working population (employed in a paid job or self-employed) [23]. The study by Toh et al. [9] found anxiety, depression, and stress at higher levels and poorer overall quality of life among essential workers other than healthcare workers.

Studies have established several factors that are connected with poorer mental health during COVID-19, including being young [15,21,26,27], being female [15,18,21,23], having higher educational level [27], being single/separated [15,27], job loss [23,24], being anxious about getting infected with COVID-19 [24,28], perceived chances of surviving [17], and adverse impact of restrictions on daily life [24]. Among the factors identified are individuals with chronic sickness and poor self-rating of health status [17], and pre-existing or previous diagnosis of depression and anxiety [23,28] and lived experiences of mental illness [15]. Furthermore, the risk of severe COVID-19 infection has been found to be higher among individuals with risky lifestyle behaviours including smoking, low levels of physical activity and obesity [29,30]. Lifestyle behaviours are strongly associated with the mental health outcomes and contribute to the negative mental health consequence of COVID-19 [31]. For instance, increased alcohol consumption was associated with increased depressive symptoms, poor mental health and mental well-being during the COVID-19 pandemic [32].

Fly-in fly-out (FIFO) work is a kind of long-distance commuting where workers are flown to worksites to temporarily stay and work over a specified period before they fly back home [33]. Over recent years, FIFO work arrangements have become more popular in the resources industry, such as onshore mining and offshore oil and gas in several countries around the world including Australia, Canada, UK and Norway [34]. In Australia, it is estimated that about 17% of persons in some regional areas are employed on FIFO work arrangements [35]. Studies pre-COVID 19 have suggested FIFO workers in the resources sector in Australia have higher prevalence of psychological distress [34], loneliness and social isolation [36,37] and suicidal risk [38], and risky levels of alcohol consumption [39] than reported in the general populace. For instance, a study has reported 28% prevalence of high to very high psychological distress among FIFO workers compared to 10.8% in the general population [40]. This suggests that FIFO workers may be at a higher risk of experiencing worse mental health problems during the COVID-19 pandemic. FIFO work involves travelling long distances to isolated areas, rotating consecutive days working and living on-site with periods at home [33]. Compared to other work arrangements and the general population, FIFO workers typically work long hours of standard 12 h shifts that may alternate between day and night shifts [41], live in provided accommodations at worksites away from their home [33,41], and work on rotation systems of for example 14 days on and 7 days leave [33], suggesting intermittent separations from families and other social support. The FIFO work arrangements are commonly practiced in the mining sector; classified as ‘essential workplace—where work cannot be done from the home’ [42], workers were exempted from stay home orders during the COVID-19 pandemics, but traveling workers were subjected to quarantines and/or self-isolations. Furthermore, being a high-risk industry, the advent of the pandemic suggests further quarantine and self-isolation on-sites, limited social activities at campsites and possible extended work periods during COVID-19 lock-downs, which may further propagate the distress, loneliness and isolation experienced by some workers, causing poorer mental health. These may also be coupled with uncertainty/fear of job losses due to the falling prices of commodities on the world market and crippling economic nature of the pandemic, which has been found to be one of the stressors among FIFO workers [34], although the Australian economic has not been badly impacted by the pandemic.

Although, employers provide support for the mental health and well-being such as on-site counselling services [43], FIFO workers could be impacted negatively due to the changes caused by the COVID-19 pandemic. However, no study to the best of our knowledge has examined their mental well-being during this pandemic. This study aimed to examine the mental well-being of FIFO workers in the mining industry during the COVID-19 outbreak restrictions, and to explore the effects of pre-existing medical conditions, lifestyle behaviours and the COVID-19 exposure and experience of related symptoms on their mental well-being.

## 2. Materials and Methods

### 2.1. Study Design and Participants

This was a cross-sectional study conducted as part of a comprehensive COVID-19 screening program initiated by a large mining company in Western Australia for FIFO workers between May to November 2020 dubbed Comprehensive health implications of Coronavirus (COVID-19) exposure in the community (CIVIC)—Fly In Fly Out Screening Study (CIVIC-FIFO). The study used convenient sampling: all FIFO workers aged 18 years and over, working on remote mining and construction sites associated with the mining company in Western Australia, were invited to take part in the study.

### 2.2. Data Collection

Data were collected online using the RedCAP research data capture tool (https://www.project-redcap.org, accessed on 5 May 2020). The URL link and a QR code to the study were sent to the workers by the company as part of the appointment email/text asking workers to present at the CIVIC-FIFO screening program. Workers interested in participating in the study, using the URL link and/or by scanning the QR code, were directed to an online participant information sheet, to consent and complete an online questionnaire. The questionnaire collected information on socio-demographic characteristics, lifestyle behaviours, medical history, COVID-19 exposure and related symptoms, and mental health and well-being. All 9301 FIFO workers taking part in the COVID-19 screening were invited, out of which 1200 workers responded and completed the questionnaire, giving a response rate of 12.9%.

### 2.3. Measurements

#### 2.3.1. Socio-Demographic Characteristics

Socio-demographic characteristics of the participants included age, gender, and ethnicity, were collected through self-report. Educational level, marital status, and occupation were not assessed, as participants were not willing to provide data on those variables.

#### 2.3.2. Lifestyle Behaviours

Smoking: Participants were asked whether they had smoked tobacco regularly for 5 days or more per week during the past year. Their responses were coded as No (0): non-smoker and Yes (1): smoker.

Alcohol consumption was measured as current intake of alcohol and the responses coded as current consumer (1), former consumer (2) and never consumed alcohol (3).

Physical activity/exercise was measured as engaging in moderate to vigorous exercises/activities in general. Vigorous activity was defined as any hard physical effort which makes you breathe much harder than normal. Participants were asked to indicate how many days per week they usually engage in moderate/vigorous exercise. Using the Center for Disease Control and Prevention (CDC) recommendation of “an equivalent mix of moderate- and vigorous-intensity aerobic activity on 2 or more days a week” for health benefits [44], responses were categorized into: 0–1 day of exercise as inadequate physical activity (0) and 2–7 days of exercise as adequate physical activity (1).

#### 2.3.3. Medical History

Participants self-reported their medical history by asking them to indicate if they have had a diagnosis of medical conditions using a checklist including hypertension, high cholesterol, been diagnosed with asthma, chronic kidney disease or kidney failure, lung disease, diabetes, cancer, immune disorders, and auto-immune disorders (e.g., lupus, coeliac, multiple sclerosis) [45] and the responses coded as ‘Yes (1)’, ‘No’(0), and ‘Unknown (0)’. A composite variable was created and categorised as; no underlying condition (0), one condition (1), and 2 or more conditions (2).

Participants were also asked to indicate whether they took any regular medication and to indicate any history of heart disease or stroke in a first-degree relative (father, mother, brother or sister age < 65 years). Responses to both items were coded ‘Yes’ (1) and ‘No’ (0).

#### 2.3.4. COVID-19 Exposure and Related Symptoms

COVID-19 exposure comprises of the experiences of COVID-19 precautionary measures including quarantine, self-isolation, testing for COVID-19 and impact of social distancing guidelines undertaken as part of measures to control the spread of COVID-19.

Travel quarantine: participants were asked if they have been placed under quarantine either due to travelling back into Australia or between states, and response coded as ‘Yes’ (1) and ‘No’ (0).

Self-isolation status: Self-isolation was measured using 4 items, each scored on a ‘Yes’ and ‘No’ responses. Participants were asked if they have (1) had to self-isolate due to being in close contact with a person who tested positive to COVID-19; (2) had to self-isolate due to awaiting COVID-19 test results; (3) had to self-isolate due to being tested positive to COVID-19 but not requiring hospitalisation; and (4) had to self-isolate due to being symptomatic but not meeting criteria for COVID-19 testing. A composite variable was created and categorised as ‘Yes’ (1): at least one experience of self-isolation and ‘No’ (0): no experience of self-isolation.

COVID-19 testing: Participants were asked if they have been tested for COVID-19 but the result was negative and response coded as ‘Yes’ (1) and ‘No’ (0).

Impact of social distancing guidelines: Participants were asked if they have been impacted negatively by social distancing guidelines and responses coded as ‘Yes’ (1) and ‘No’ (0).

Common COVID-19 related symptoms: Participants self-reported experiences of any of the common signs and symptoms related to COVID-19. Participants were asked to indicate if they have had any of the signs or symptoms over the last two weeks using a checklist including fever, cough, throat, runny nose, muscle and joint pains, fatigue, shorten of breath, nausea/vomiting, diarrhea, and headache [46]. Responses were coded as ‘Yes’ (1), ‘No’ (0) and ‘’Unknown (0), and a composite variable was created and categorized as none: no symptoms (0), one symptom (1) and 2 or more symptoms (2).

#### 2.3.5. Mental Well-Being

Mental well-being was assessed employing the Short Warwick–Edinburgh Mental Well-being Scale (SWEMWBS) [47]. The SWEMWBS assesses mental well-being over the past 2 weeks and has been validated for measuring mental well-being in the general population [47,48] and used in the Australian adult population of university teachers [49]. This scale has shown high level of internal reliability (Cronbach’s α = 0.84) [50]. The SWEMWBS tool uses 7 positively-worded items (e.g., *I’ve been feeling optimistic about the future, I’ve been feeling relaxed, I’ve been feeling useful, I’ve been thinking clearly*), scored on a 5-point Likert scale: 1 (none of the time) to 5 (all of the time). The overall raw item-scores for each individual were calculated by adding each item score and the total converted to metric scores guided by the conversion table of the SWEMWBS [47], as a linear transformation of the raw scores is recommended for parametric tests [47]. SWEMWBS scores ranged from 7 to 35 with a higher score indicating the highest possible mental well-being.

### 2.4. Data Analysis

Data were analysed using STATA version 13 (StataCorp LP, College Station, TX, USA). Descriptive statistics were assessed for continuous and categorical variables. One-way analysis of variance (ANOVA) was conducted to examine the differences in mental well-being across the different socio-demographic, lifestyle behaviours, history of medical conditions, COVID-19 exposure measures, and COVID-19 related symptoms. Post hoc comparisons were conducted for significant variables using Bonferroni t-tests to examine the differences between age, placement under travel quarantine, self-isolation, impacted by social distancing guidelines, and experience of common COVID-19 related symptoms groups. Further, multiple linear regression analysis was conducted to examine the association between socio-demographic, lifestyle behaviours, history of medical conditions, COVID-19 exposure measures, and COVID-19 related symptoms in relation to the mental well-being of FIFO workers during the early restriction periods of COVID-19 pandemic in Australia. The level of significance was set at *p* < 0.05.

## 3. Results

### 3.1. Characteristics of Study Respondents

One thousand two hundred (1200) FIFO workers responded to the survey; out of which 358 were excluded due to missing data and a final sample of 842 was included in the study. The mean age of the study participants was 44.1 ± 11.8 years, with most (74.7%) aged 35 years and above. The majority of the workers were male (82.4%) and were of Caucasian/White ethnic background (80.0%) (see Table 1).

The majority of FIFO workers were current alcohol drinkers (76.2%) with 33% of workers indicating that they smoked. Most of the workers (65.6%) engaged in adequate moderate to vigorous exercises per week. Concerning medical history, 41.5% of the workers indicated that they had one or more chronic conditions; commonly high cholesterol levels (13.7%), hypertension (13.2%), asthma (12.7%), cancer (10.6%), and diabetes (8.2%). About 28% of the workers were on regular medications, and 15.4% of them reported family history of heart diseases in their first line relations (see Table 1).

### 3.2. Mental Well-Being of FIFO Workers

The mean SWEMWBS scores among workers was 24.8 ± 4.7, (range 7–35). A one-way ANOVA revealed significant differences in SWEMWBS scores by age, being placed under travel quarantine, undertaken self-isolation, impacted by social distancing guidelines, and experienced common COVID-19 related symptoms (see Table 1). Bonferroni post hoc tests (see Supplementary Table S1) revealed FIFO workers aged 55 years and above (26.6 ± 4.7) had significantly higher SWEMWBS scores compared to those aged less than 25 years (24.3 ± 5.2), 25–34 years (23.8 ± 4.8), 35–44 years (24.2 ± 4.8) and 45–55 years (24.8 ± 4.0), (*p* < 0.001). However, significantly lower scores were found in workers who had undertaken a travel quarantine (23.6 ± 4.6) vs. 24.9 ± 4.7), *p* = 0.047), self-isolation (23.3 ± 4.8 vs. 24.9 ± 4.7, *p* = 0.027) and were negatively impacted by social distancing guidelines (24.0 ± 4.0 vs. 25.1 ± 4.9, *p* = 0.002) compared to those who have not. FIFO workers who experienced one (23.6 ± 4.5) or 2 or more (22.8 ± 3.6) COVID-19 related symptoms also had significantly lower scores compared to those who experienced no symptoms (25.1 ± 4.8) (*p* < 0.001).

In a multiple linear regression analysis, workers aged 55 years or more compared to less than 25 years (β = −2.27, 95%CI = −3.96–−0.59), 25–34 years (β = −2.86, 95%CI = −3.89–−1.83), 35–44 years (β = −2.03, 95%CI = −3.00–−1.05) and 45–54 years (β = −1.95, 95%CI = −2.87–−1.04) compared to those 55 years or more had low scores on mental well-being. Similarly, workers who had undertaken a travel quarantine (β = −1.42, 95%CI = −2.75–−0.09) compared to those who have not, and workers who had 2 or more COVID-19 related symptoms compared to those who had no symptoms (β = −1.97, 95%CI = −3.09–−0.86) had lower scores on mental well-being (see Table 2).

## 4. Discussion

This study examined FIFO workers’ mental well-being during the COVID-19 pandemic and its relationships with history of chronic conditions, experiences of common COVID-19 related symptoms and COVID-19 related exposures in the mining industry in Western Australia.

Findings showed COVID-19 and initial restrictions may have not significantly negatively impacted on the mental well-being of FIFO workers. The average SWEMWBS scores for mental well-being of FIFO workers (24.8 ± 4.7) during the COVID-19 pandemic were higher than the population norms (23.6 ± 3.9) [51] and that reported in university teachers samples (21.5 ± 4.1) in Australia [49]. This finding is in discordant to the findings of studies suggesting higher levels of negative emotions, depression, anxiety and stress and poor mental health associated with COVID-19 related measures in the general population in Australia [9,15,23] and other countries [16,21,52]. Though the current results may suggest FIFO workers were able to cope with the disruption and stress of COVID-19 related measures, the differences observed could reflect the disparities in measurements, the periods at which studies were conducted and the samples as the current sample were mostly men who are generally less likely to experience internalized disorders including depression and anxiety, except for externalize disorders including aggression [53].

Older FIFO workers aged 55 years and over reported better mental well-being as compared to younger workers aged below 55 years; against the backdrop that increased age is associated with COVID-19 mortality [54]. This current finding supports findings of previous studies where younger people were found to experience poor mental health during COVID-19 pandemic in Australia [15] and other countries [16,21,26,27]. Mental health has been indicated to be of increased concern among young people [55], with environmental experiences such as social inequality [56] among others indicated to play an important role in worsening mental health in this population group. In Australia, a national survey has indicated equity and discrimination as one of the major concerns expressed by young people [55]. Furthermore, young people often report problems with coping with stress [55] whereas older age is associated with better emotional stability and higher positive emotional well-being [57]. As such, younger people may find it more difficult to cope with stress associated with the COVID-19 pandemic, compared to older people who may have developed adaptive skills due to the experiences of previous crises [16]. Again, younger workers may experience negative emotions due to being away from their young families/children at home and also may have additional childcare responsibilities and/or increased financial strain during the pandemic [15]. In addition, the older FIFO workers may have worked FIFO for longer periods and adjusted to the FIFO lifestyle, had more life experiences and developed the emotional resilience and coping skills for stressful events. The current finding suggests the need for younger FIFO workers to be monitored and provided with the support to cope with stresses associated with the COVID-19 pandemic and the negative mental health issues that may arise during this period.

The study found workers who had faced travel quarantines had poor mental well-being than those who had not faced travel quarantines. Consistent with the findings of studies from other populations elsewhere where individuals facing travel quarantines were associated increased risk of depression, anxiety, stress [27], suicidal ideation and self-harm [58], and decline mental health well-being [59]. Furthermore, experiencing common COVID-19 related symptoms was associated with poor mental well-being. Consistent with the findings of a previous study, which found higher level of anxiety, depression, and stress in individuals who experienced symptoms similar to the symptoms of COVID-19 among the general population in the United States [60]. Experiencing symptoms consistent with the symptoms of COVID-19 may increase the anxiety of being infected or getting loved ones infected with COVID-19 as well as not being able to work. Additionally, as per the public health guidelines, one would be required to self-isolate or quarantine, observe social/physical distancing and/or get tested for COVID-19 [61], all of which have been indicated to cause frustration and anxiety [14] and impact on one’s social support systems, and coping mechanisms that positively impact on mental health [13].

There was no observed significant association between previous medical history and mental well-being in our study. This is in contrast with the observations made in other studies elsewhere in the general population where individuals with history of chronic illness/pre-existing health conditions typically reported poorer mental health outcomes such as stress, anxiety and depression [17,60] and lower mental well-being [52]. Having pre-existing health conditions can predispose individuals to severe or deadly COVID-19 infections [60], and as such, could increase the anxiety around getting infected and perceived survival risk when infected, among individuals with pre-existing conditions. Such individuals, as part of protecting themselves from getting infected, are most likely to suffer reduced social interactions significant to promote mental health [13] due to the social and physical distancing guidelines. Furthermore, precautionary COVID-19 exposure measures including self-isolation, impact of social distancing guidelines and testing for COVID-19, and lifestyle behaviours including alcohol intake, smoking and physical activity were found not to be associated with mental well-being in FIFO workers. Findings inconsistent to our study have been documented in other studies e.g., [15,32,62,63]. FIFO workers work in stressful environments and on job arrangements that involve separation from partners and/or families [64], and as such, workers who are able to develop the skills to cope/adapt or having built a resilience to the work stresses are able to remain in the workforce [65]. This could suggest that most FIFO workers in our study may have developed the emotional stability and resilience to be able to cope with stresses of COVID-19 pandemic and related control measures.

The findings of this study could contribute to the development of informed interventions during this and future pandemics and other future programs aimed at promoting mental health and well-being among FIFO and mining workers in the mining industry. In this regard, public health managers and organisations should support interventions that identify and prioritise vulnerable groups to guarantee their access to the needed support. Such interventions could include the monitoring and provision of appropriate and sustained support to cope with stresses associated with the COVID-19 pandemic and the negative mental health issues that may arise during COVID-19 pandemic, its restriction measures and the subsequent economic disruptions. The findings of this study are also suggestive of the need for public health and organizations to provide prompt and adequate information about the COVID-19 infection and its accompanying public health measures, such as quarantine or self-isolations. Consistent and adequate information on the COVID-19 infection could minimize the anxiety that may surround experiences of symptoms that could be consistent with COVID-19 infections. Furthermore, such information provided in a prompt manner to persons under quarantine will enable better understanding of the importance of being quarantined and minimize the anxiety of being infected or infecting loved ones [14]. The negative mental health consequences of quarantine measures highlighted in this study also underpin the need for public health mangers and organizations to limit the length of quarantine periods, and provide persons with environments that reduce the sense of isolation and improve communication between persons and their families and social networks during quarantines. This has previously been shown to minimize the associated negative consequences of quarantine and improve mental health in other setting, and is likely extendable to the FIFO community [14].

This study is the first to characterise the mental well-being of FIFO mining workers during the restrictions of the COVID-19 pandemic in Western Australia. However, some limitations of this study are acknowledged to guide the interpretation of our findings. First, the study employed cross-sectional design and techniques to collect data; as such, any causal interpretation of the findings is limited. The study also relied on participants’ self-reported data which could not be verified and could therefore be subjected to under- or over-estimation of the levels of the study parameters. The study is also limited by small sample survey and self-selection bias: the study relied on non-probability convenient sampling techniques to recruit participants, thus could have resulted in a non-representative sample of the FIFO workers, where workers who were either positively or negatively impacted by COVID-19 choosing to participate in the study or otherwise. Data on participants’ educational level, marital status, and occupation were excluded as participants were not willing to provide data on, leading to several loss of data, as such, these variables could not be adjusted for in the multiple linear regression model though they have been documented to be associated with mental health outcomes during COVID-19 [27]. The measure of physical activity may not provide a more precise estimate of typical moderate/vigorous physical activity levels, as the item used did not specify or collect data on the number of minutes spent per day to engage in physical activity. There were also low reported cases of COVID-19 in Western Australia compared to other Australian states like Victoria and New South Wales and reported in other countries. Furthermore, there was also no reported case of COVID-19 among the study population during the course of the study period.

## 5. Conclusions

In conclusion, there was positive/better mental well-being during COVID-19 related restrictions in this sample of FIFO workers in Australia; suggesting FIFO workers may have been able to cope with the disruption and stress of COVID-19 and related early restrictions. However, workers who were younger, placed under travel quarantine and experienced symptoms consistent with the common symptoms of COVID-19 had poor mental well-being. Knowing and admitting the negative emotions and distress experiences among this work groups may help in providing suitable support and interventions to help lessen these negative experiences. This study identified vulnerable groups of FIFO workers that need to be monitored and provided with the needed and sustained support to cope with stresses associated with the COVID-19 pandemic and the negative mental health issues that may arise during COVID-19 pandemic and its subsequent restriction measures.

## Figures and Tables

**Table 1:** Background characteristics of FIFO workers by mental well-being scores (*N* = 842).

Parameters	Frequency	Percent	Mental Well-Being Scores, m(sd)	*f* Statistics	*p*-Value
Age in years					
≤24	39	4.6	24.3 (5.2)		<0.001 *
25–34	174	20.7	23.8 (4.8)	F(4, 837) = 10.22	
35–44	207	24.6	24.2 (4.8)		
45–54	235	27.9	24.8 (4.0)		
≥55	187	22.2	26.6 (4.7)		
Sex					
Male	694	82.4	24.8 (4.7)	F(1, 840) = 0.06	0.802
Female	148	17.6	24.7 (4.7)		
Ethnicity					
Caucasian/White	680	80.8	24.7 (4.5)	F(8, 833) = 2.27	0.132
Others	162	7.1	25.3 (5.4)		
Place under quarantine					
Yes	54	6.4	23.6 (4.6)	F(1, 840) = 3.95	0.047 *
No	788	93.6	24.9 (4.7)		
Undertaken self-Isolation					
Yes	46	5.5	23.3 (4.8)	F(1, 840) = 4.91	0.027 *
No	796	94.5	24.9 (4.7)		
Tested for COVID-19 but the result was negative					
Yes	328	39.0	24.8 (4.8)	F(1, 840) = 0.03	0.861
No	514	61.0	24.7 (4.5)		
Impacted by social distancing guidelines					
Yes	241	28.6	24.0 (4.0)	F(1, 840) = 10.02	0.002 *
No	601	71.4	25.1 (4.9)		
Any underlying condition					
None	493	58.5	24.7 (4.9)		
One condition	216	25.7	25.1 (4.6)	F(2, 839) = 0.59	0.564
2 or more conditions	133	15.8	24.7 (3.9)		
Family history of heart disease					
Yes	130	15.4	25.0 (4.5)	F(1, 840) = 0.30	0.583
No	712	84.6	24.8 (4.7)		
Taking regular medication					
Yes	239	28.4	24.8 (4.5)	F(1, 840) = 0.00	0.983
No	603	71.6	24.8 (4.8)		
COVID-19 related symptoms					
None	689	81.8	25.1 (4.8)		
One symptom	75	8.9	23.6 (4.5)	F(2, 839) = 11.62	<0.001 *
2 or more symptoms	78	9.3	22.8 (3.6)		
Smoking status					
Yes	280	33.2	24.7 (4.4)	F(1, 840) = 0.06	0.803
No	562	66.8	24.8 (4.8)		
Alcohol intake					
Current user	642	76.2	24.7 (4.5)		
Former user	106	12.6	24.7 (4.7)	F(2, 839) = 2.11	0.122
Never used	94	11.2	25.7 (5.8)		
Moderate/vigorous exercise					
Inadequate	290	34.4	24.6 (4.8)	F(1, 840) = 0.64	0.424
Adequate	552	65.6	24.9 (4.6)		

* Significant at *p* < 0.05; m(sd) = mean scores (standard deviation).

**Table 2:** Linear regression of the correlate of mental well-being among FIFO workers.

Parameters	β	SE	95%CI		*p*-Value
			Lower	Upper	
Age in years					
≥55	Ref				
45–54	−1.95	0.46	−2.87	−1.04	<0.001 *
35–44	−2.03	0.50	−3.00	−1.05	<0.001 *
25–34	−2.86	0.53	−3.89	−1.83	<0.001 *
≤24	−2.27	0.86	−3.96	−0.59	0.008 *
Sex					
Male	Ref				
Female	0.14	0.43	−0.71	0.99	0.744
Ethnicity					
Caucasian/White	Ref				
Other	0.46	0.43	−0.38	1.30	0.283
Place under quarantine					
No	Ref				
Yes	−1.42	0.68	−2.75	−0.09	0.037 *
Undertaken self-Isolation					
No	Ref				
Yes	−0.11	0.74	−1.57	1.35	0.878
Tested for COVID-19 but the result was negative					
No	Ref				
Yes	0.28	0.34	−0.38	0.93	0.408
Impacted by social distancing guidelines					
No	Ref				
Yes	−0.73	0.37	−1.46	0.001	0.051
Any underlying condition					
None	Ref				
One condition	0.56	0.41	−0.25	1.37	0.178
2 or more conditions	−0.20	0.53	−1.24	0.85	0.712
Family history of heart disease					
No	Ref				
Yes	−0.39	1.38	−3.10	2.32	0.779
Taking regular medication					
No	Ref				
Yes	−0.54	0.42	−1.36	0.28	0.196
COVID-19 related symptoms					
None	Ref				
One symptom	−0.92	0.58	−2.06	0.21	0.109
2 or more symptoms	−1.97	0.57	−3.09	−0.86	0.001 *
Smoking status					
No	Ref				
Yes	−0.22	0.35	−0.91	0.45	0.529
Alcohol intake					
Never used	Ref				
Former user	−0.94	0.67	−2.26	0.39	0.165
Current user	0.69	0.54	−1.76	0.37	0.203
Moderate/vigorous exercise					
Inadequate	Ref				
Adequate	0.37	0.34	−0.30	1.04	0.282

* Significant at *p* < 0.05, ref = reference for category; CI = confidence interval.

## Data Availability

The data presented in this study are not publicly available but are available from the corresponding author on reasonable request.

## Supplementary Materials

The following are available online at www.mdpi.com/article/10.3390/ijerph182212264/s1, Table S1: Bonferroni post hoc comparison of mental well-being between independent variable groups.
