# Supplementary material for: Mental Well-Being during COVID-19: A Cross-Sectional Study of Fly-In Fly-Out Workers in the Mining Industry in Australia
Source: Int J Environ Res Public Health. 2021 Nov 22;18(22):12264. doi: 10.3390/ijerph182212264 (PMC8620700; doi:10.3390/ijerph182212264)
Supplement: Supplementary file 1 [file ijerph-18-12264-s001.zip › ijerph-1469726-supplementary.pdf]

Supplementary Material

Table S1. Bonferroni post hoc comparison of mental well-being between independent variable groups.

Characteristics	coefficient	*p*-value
**Age group**		
24 vs 25-34	-0.502	1.000
24 vs 35-44	-0.122	1.000
24 vs 45-54	0.467	1.000
24 vs 55+	2.290	0.048
25-34 vs 35-44	0.380	1.000
25-34 vs 45-54	0.969	0.354
25-34 vs 55+	2.792	<0.001*
35-44 vs 45-54	0.589	1.000
35-44 vs 55+	2.412	<0.001*
45-54 vs 55+	1.823	0.001*
**Travel quarantine**		
No vs Yes	-1.311	0.047*
**Self-isolation**		
No vs Yes	-1.575	0.027*
**Impact by social distance guidelines**		
No vs Yes	-1.128	0.002*
**COVID-19 related symptoms**		
None vs one symptom	-1.530	0.020*
None vs 2 or more symptoms	-2.348	<0.001*
One symptom vs 2 or more symptoms	-0.818	0.828

*significant at p<0.05.
